# Role of Perindopril in Mitigating Doxorubicin’s Vascular Toxicity in a Rat Model

**DOI:** 10.1007/s12012-026-10092-0

**Published:** 2026-01-27

**Authors:** Anna Marada, Tibor Stračina, Filip Marhefka, Lucie Šůstková, Jaroslav Nádeníček, Jindra Smutná, Peter Scheer, Jana Hložková, Michal Hendrych, Christian Studenik, Hana Paulová, Marie Nováková

**Affiliations:** 1 Department of Physiology, Faculty of Medicine, Masaryk University, Kamenice 5, Brno, 625 00 Czech Republic; 2 Department of Biochemistry, Faculty of Medicine, Masaryk University, Brno, Czech Republic; 3 Department of Pharmacology and Toxicology, Faculty of Pharmacy, Masaryk University, Brno, Czech Republic; 4 International Clinical Research Center, St. Anne’s University Hospital Brno and Faculty of Medicine, Masaryk University, Brno, Czech Republic; 5 First Department of Pathology, Faculty of Medicine, Masaryk University and St. Anne’s University Hospital Brno, Brno, Czech Republic; 6 Division of Pharmacology and Toxicology, Department of Pharmaceutical Sciences, University of Vienna, Vienna, Austria

**Keywords:** Doxorubicin, Vascular toxicity, Perindopril, Isolated aortic ring, Rat, 4-hydroxy-2-nonenal

## Abstract

Doxorubicin (DOX), a widely used anthracycline in cancer therapy, is associated with significant cardiovascular toxicity. While its cardiotoxic effects are well documented, the mechanisms and prevention of DOX-induced vascular toxicity remain insufficiently explored. Angiotensin-converting enzyme inhibitors (ACEIs), such as perindopril (PER), are commonly used in cardiovascular disease management and may offer vascular protection during chemotherapy. Female ovariectomized Wistar rats were treated with *i.v.* DOX and/or *p.o.* PER over five weeks. Cardiac and vascular function were assessed using high-frequency ultrasound and ECG. Vascular reactivity was evaluated in isolated aortal rings using phenylephrine (PE), acetylcholine (ACh), L-N-Nitro arginine methyl ester hydrochloride (L-NAME), and verapamil (VER). Oxidative stress was assessed via plasma 4-hydroxy-2-nonenal (4-HNE) levels, and structural changes were monitored through intima-media thickness (IMT) measurements. DOX administration significantly impaired vascular reactivity, as evidenced by increased contractile responses to PE and reduced endothelium-dependent relaxation. These functional alterations were accompanied by elevated plasma 4-HNE levels, indicating enhanced oxidative stress. Co-treatment with PER preserved vascular responsiveness, reduced contractile tension, and significantly lowered 4-HNE concentrations. Structurally, IMT increased in control and PER-only groups, likely due to post-ovariectomy remodelling, while DOX-treated groups showed no IMT progression. PER co-treatment appeared to stabilize IMT values. PER mitigates DOX-induced vascular toxicity, likely through endothelial protection and reduction of oxidative stress. These findings support the potential use of ACEIs as prophylactic agents in patients undergoing anthracycline-based chemotherapy and highlight the need for further translational studies in cardio-oncology.

**Graphical Abstract:**

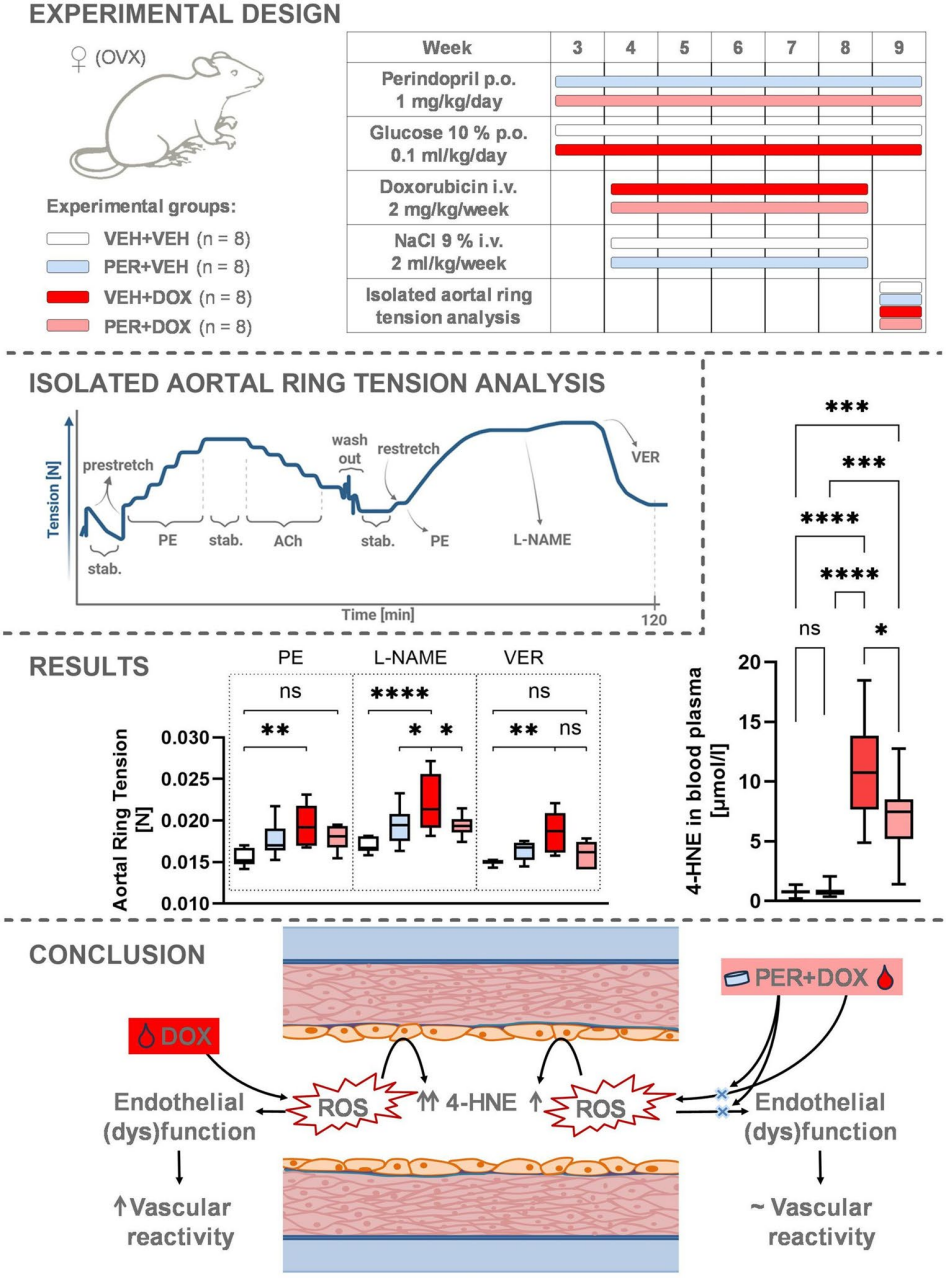

**Supplementary Information:**

The online version contains supplementary material available at 10.1007/s12012-026-10092-0.

## Introduction

According to the World Health Organization, cardiovascular diseases and malignancies are the leading causes of morbidity and mortality globally [1–3]. Their coexistence, particularly in elderly and polymorbid patients, represents a significant clinical challenge. Many cancer therapies, including but not limited to anthracyclines, are associated with cardiovascular side effects; while pre-existing cardiovascular comorbidities can significantly limit the safety and efficacy of oncological treatment [4, 5]. Moreover, the psychological stress associated with a cancer diagnosis often triggers activation of the sympathoadrenal system. Persistent sympathetic overactivity may contribute to the development or progression of cardiovascular disease. This complex interplay between oncological treatment, physiological stress responses, and cardiovascular risk highlights the need for integrated cardio-oncology strategies, particularly in managing drug interactions and preserving long-term cardiovascular health [1–5].

DOX, a widely used anthracycline antibiotic, remains a cornerstone of chemotherapy for a broad range of solid tumours and haematological malignancies. Its antitumour efficacy is mediated through multiple mechanisms, including DNA intercalation, inhibition of topoisomerase II, and the generation of reactive oxygen species (ROS) [6, 7]. Among its many applications, DOX is a key component of treatment regimens for aggressive cancers such as triple-negative breast cancer (TNBC), where therapeutic options are limited and cytotoxic chemotherapy remains the standard of care [8–14].

Despite its clinical utility, DOX is associated with significant dose-dependent cardiotoxicity and vascular side effects, which can compromise long-term outcomes, particularly in cancer survivors. As survival rates improve, the delayed cardiovascular consequences of chemotherapy have become a growing concern. This has led to the emergence of cardio-oncology as a discipline focused on minimizing cardiovascular risks without compromising oncological efficacy. Within this context, strategies to mitigate DOX-induced vascular toxicity are of increasing interest, especially in patients with pre-existing cardiovascular risk factors or those receiving long-term or high-dose anthracycline therapy [15–20].

Proposed cardiotoxic mechanisms of DOX include, among others, interaction with topoisomerase 2β, DNA damage, apoptosis, autophagy, oxidative stress, and renin-angiotensin-aldosterone system (RAAS) dysregulation [7, 15, 17, 21, 22]. Oxidative stress plays a central role, characterized by excessive generation of ROS and diminished antioxidant defences in cardiomyocytes. This leads to mitochondrial dysfunction, iron metabolism disruption, endoplasmic reticulum stress, and membrane damage through lipid peroxidation. Additional mechanisms include nitric oxide (NO) imbalance, endothelial dysfunction, ATP depletion, calcium dysregulation, inflammatory mediator release, and activation of the ubiquitin-proteasome system – all contributing to cardiovascular damage [15–17, 21, 23, 24]. RAAS dysregulation has emerged as a promising target for cardioprotection. It plays a critical role in cardiovascular homeostasis, and its disruption is concerned in development of hypertension, heart failure, and even cancer progression. DOX may alter RAAS activity by modifying gene expression and enhancing angiotensin II signalling; however, it remains unclear whether these effects are systemic or localized to the myocardium [18, 21, 25–31].

Clinically, DOX-induced cardiotoxicity is characterized by a broad spectrum of clinical symptoms, from early subcellular changes to manifest left ventricular dysfunction. Even a single dose can initiate myocardial injury, detectable via ECG or echocardiography (ECHO), and may lead to acute cardiomyopathy or subclinical dysfunction that progresses to heart failure over time. These risks underscore the urgent need for strategies that mitigate cardiotoxicity during anthracycline therapy [15–20, 32].

While DOX-induced cardiotoxicity has been extensively studied [6, 15, 16, 20, 23, 24, 27–29, 32–35], its vascular effects remain comparatively underexplored [36–41]. Clinical and experimental evidence suggests that DOX administration contributes to systemic vasculitis, vascular remodelling, arterial stiffness, hypertension, and atherosclerosis [39, 42]. These vascular complications may emerge during treatment or manifest later [40], yet they are rarely monitored in routine oncological care. Unlike cardiac assessments (e.g., ECG, ECHO, cumulative dose tracking), vascular evaluation, such as IMT or arterial stiffness indices, is not standard practice, often overshadowed by the urgency of cancer treatment.

The mechanisms underlying DOX-induced vascular toxicity are not fully elucidated. A central question remains whether the damage is primarily endothelium-dependent, endothelium-independent, or a combination of both. Most studies report impaired endothelial function, including structural lesions and reduced endothelium-dependent relaxation. Some also suggest direct effects on vascular smooth muscle (VSM); however, the results of such studies are inconsistent [36–38, 40, 41, 43, 44]. These discrepancies likely stem from variations in experimental design, including species differences, administration routes, exposure duration, dosage, and evaluation methods.

As previously noted, modulation of the RAAS has been widely explored as a strategy to reduce DOX-induced cardiotoxicity [21, 25–29]. ACEIs, commonly prescribed for hypertension and heart failure, have shown cardioprotective effects in this context. However, their potential to mitigate DOX-induced vascular toxicity remains largely under-investigated. Despite the widespread clinical use of ACEIs, studies examining their concurrent administration with DOX and the resulting impact on vascular integrity are scarce [18, 21, 38]. Currently, there are no specific guidelines addressing vascular protection during cancer therapy. In clinical practice, cardiovascular comorbidities in oncology patients are typically managed according to standard cardiovascular treatment protocols [18, 19, 45, 46], without adjustments for the added vascular risks posed by chemotherapy.

This lack of targeted research – both in clinical settings and preclinical models – highlights the urgent need for comprehensive studies using translationally relevant models. Addressing this gap will require close collaboration between clinicians (e.g., oncologists, cardiologists, internists) and scientists in preclinical research. Such interdisciplinary efforts are essential to translate emerging mechanistic insights into effective, evidence-based strategies for vascular protection in cancer patients.

Although the cardiotoxic effects of DOX are well recognized, its impact on the vascular system remains insufficiently addressed in both research and clinical practice. PER, a commonly used ACEI also in cancer patients [45], has demonstrated cardioprotective and endothelial-stabilising properties, making it a potential candidate for vascular protection during chemotherapy [21, 27]. However, its role in mitigating DOX-induced vascular toxicity has not been thoroughly investigated. Therefore, the aim of this study was to evaluate whether PER can attenuate vascular damage caused by DOX. We hypothesize that PER, through modulation of the RAAS and oxidative stress, can reduce endothelial dysfunction and vascular alterations associated with DOX exposure.

## Methods

### Chemicals

Isofluranum (Aerrane, Baxter SA, Switzerland), Heparinum natricum (Heparin Léčiva, Zentiva, Czech Republic; syringe solution), Glucosum 5% (Glucose 5 B. Braun, Germany; intravenous solution for infusion), Natrii chloridum 0.9% (Sodium chloride B. Braun, Germany; intravenous solution for infusion), Ketoprofenum (Ketonal 100 mg amp., Sandoz, Slovenia; syringe solution), Perindoprilum argininum (Prestarium Neo, 5 mg, Les Laboratoires Servier, France; dispersible tablets), Doxorubicinum hydrochloridum (Doxorubicin hydrochloride, Tocris Bioscience, USA; powder), Krebs-Henseleit solution (K-H sol.; in [mM]: NaCl 118.07, KCl 4.83, KH_2_PO_4_ 1.00, MgSO_4_ 1.20, NaHCO_3_ 27.14, CaCl_2_ 2.49, glucose 10.09; pH 7.35–7.40; 37 °C; all chemicals were purchased from Sigma-Aldrich, Germany), K-H sol. saturation with 95% O_2_ + 5% CO_2_ (SIAD Czech spol. s.r.o., Czech Republic), Verapamilum hydrochloridum (Verapamil hydrochloride, Sigma-Aldrich, powder), Phenylephrini hydrochloridum (Phenylephrine hydrochloride, Sigma-Aldrich, Germany; powder), Acetylcholini chloridum (Acetylcholine chloride, Sigma-Aldrich, Germany; powder), Nω-Nitro-L-arginini methylesteri hydrochloridum (L-NAME hydrochloride, Sigma-Aldrich, Germany; powder), 10% Neutral buffered formalin (formaldehyde 4%, pH 7.2–7.4, DIAPATH S.p.A., Italy).

### Animal Model

All animal experiments were carried out according to the recommendations of the European Community Guide for the Care and Use of Laboratory Animals. The experimental protocol (No. MSMT-5763/2024-3) was approved by the Committee for Ensuring the Welfare of Laboratory Animals of Masaryk University and licensed by the Ministry of Education, Youth and Sports of the Czech Republic. The animals were housed at the Animal Breeding and Experimental Facility, Faculty of Medicine, Masaryk University, in temperature-, pressure-, and humidity-controlled environment, with light cycle 12/12 (light/dark).

**Fig. 1:**
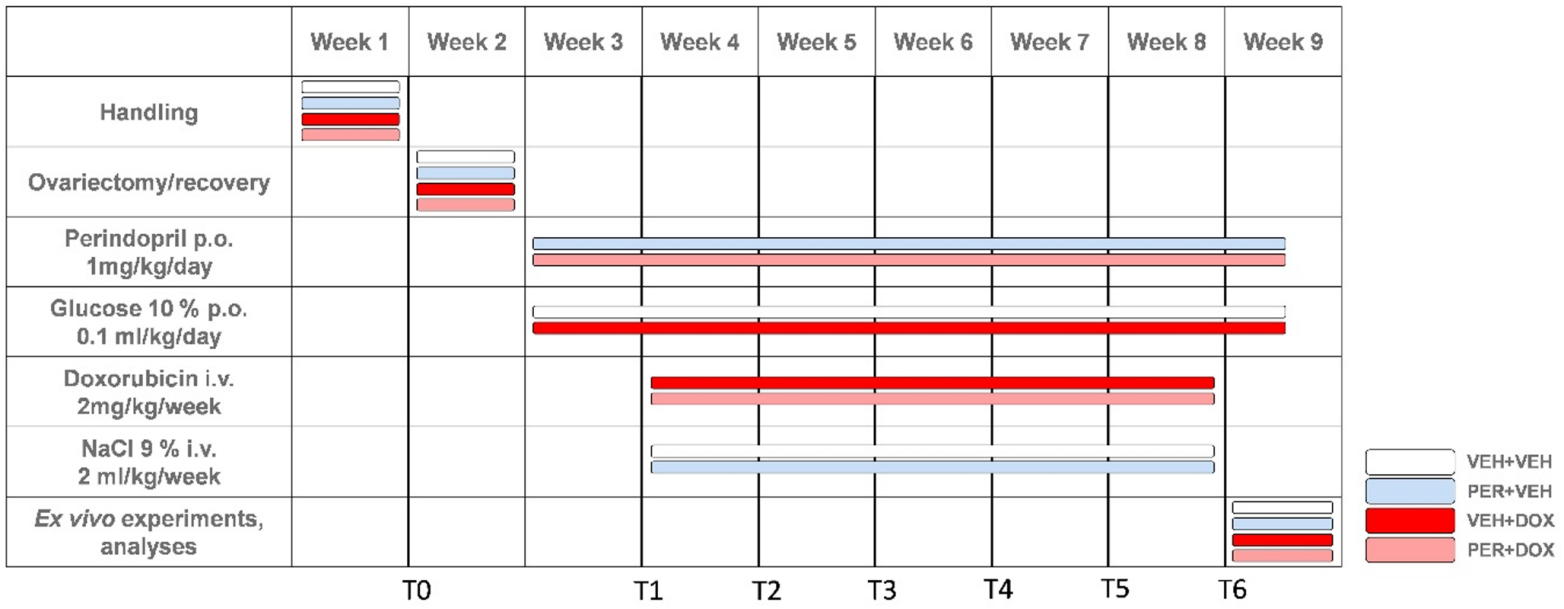
Timeline of the Experiment. The figure illustrates the schedule of cardiovascular parameters measurements and treatment administration across experimental groups: VEH + VEH (negative controls), PER + VEH (perindopril-treated rats), VEH + DOX (doxorubicin-treated rats), PER + DOX (simultaneously perindopril- and doxorubicin-treated rats). Time points T0–T6 represent key stages of the experiment: T0 – Baseline cardiovascular measurements before ovariectomy and treatment initiation; T1 – Cardiovascular measurements after one week of *p.o.* PER/VEH administration and immediately prior to the first *i.v.* DOX/VEH administration; T2–T5 – Weekly cardiovascular measurements conducted before each *i.v.* DOX/VEH administration (four consecutive weeks); T6 – Final cardiovascular measurements before experiment termination and sample collection

A total of 32 female Wistar rats (body weight 269.4 ± 14.2 g) were included in the study. At the age of 10 weeks, rats were randomly divided into 4 groups: VEH + VEH (*n* = 8, body weight 271.0 ± 22.5 g; negative controls), PER + VEH (*n* = 8, body weight 264.4 ± 16.1 g; PER-treated rats), VEH + DOX (*n* = 8, body weight 270.4 ± 5.8 g; DOX-treated rats), PER + DOX (*n* = 8, body weight 271.6 ± 7.3 g; simultaneously PER- and DOX-treated rats). All animals were fed with standard diet in pellets (Altromin, Germany). Food and water were accessible *ad libitum*. The animals were handled for a week to adapt to daily manipulation (handling phase; Fig. 1) and their general health status was evaluated regularly.

### Ovariectomy

After one week of handling, bilateral ovariectomy was performed under deep isoflurane anaesthesia (5% induction, 2.5% maintenance). Post-surgical pain was treated with ketoprofen (5 mg/kg) administered intraperitoneally once a day for 1–3 consecutive days after the surgery. Ovariectomised rats were housed individually for 3 days after the surgery to avoid possible contamination and wound reopening. Animals were allowed to fully recover for 7 days (recovery phase; Fig. 1).

### Peroral and Intravenous Substance Administration

One week after ovariectomy, *p.o.* treatment with PER (PER + VEH and PER + DOX group; 1 mg/kg/day) or vehiculum (glucose 10%; VEH + VEH and VEH + DOX group; equivalent volume of 0.1 ml/kg/day) was initiated. Substances were administered once a day at the same daytime (10:00–11:00 a.m.) for 40 consecutive days. Peroral treatment was finished a day before the experiment termination.

A week after *p.o.* treatment initiation, *i.v.* treatment with DOX (VEH + DOX and PER + DOX; 2 mg/kg/week) or vehiculum (NaCl 0.9%; VEH + VEH and PER + VEH group; equivalent volume of 2 ml/kg/week) was initiated. Intravenous treatment was performed as a single bolus dose administered into the lateral tail vein under deep isoflurane anaesthesia once a week for five consecutive weeks (Fig. 1).

During the experimental period, one animal from the VEH + DOX group developed severe local adverse effects at the site of *i.v.* DOX administration. The severity of these effects reached the predefined humane endpoint criteria established prior to study initiation. Accordingly, the animal was humanely euthanized and excluded from all subsequent analyses.

### Echocardiography

To assess cardiac function and arterial wall structure, high-frequency ultrasound imaging was performed at two time points: prior to ovariectomy (T0) and one day after the final dose of DOX (T6; Fig. 1). Ultrasound imaging was performed at the Department of Pharmacology and Toxicology, Faculty of Pharmacy, Masaryk University, Brno. The transport time did not exceed 20 min, and the animals were allowed to acclimate before the start of anaesthesia as well as after awakening. Imaging was conducted using a dedicated small-animal ultrasound system (Vevo 2100, FUJIFILM VisualSonics Inc., Toronto, Canada). Animals were deeply anaesthetised with isoflurane (2.5% for induction, 1–1.5% for maintenance) and positioned on a heated platform. The neck and thoracic regions were shaved to allow optimal probe contact.

Cardiac function was evaluated by transducer MS-201 in the parasternal long-axis view, capturing both systolic and diastolic phases of the left ventricle. Fractional shortening was calculated as a measure of systolic performance. For vascular assessment, the common carotid arteries on both sides were imaged by transducer MS-550 S in longitudinal view. IMT was measured at six independent points (three from each side) per animal, and the mean value was used for analysis.

### ECG Recording and Analysis

Animals were anaesthetised using isoflurane (5% induction, 2% maintenance) and fixed to the heated pad. One-lead ECG was recorded using needle electrodes fixed subcutaneously on the chest. The signal was amplified using Bio Amp amplifier (1 kHz sampling rate, 10 mV range; AD Instruments Ltd., CO, USA). The ECG waveform was recorded for 80 s using PowerLab 16/35 and LabChart 8 Pro software (AD Instruments Ltd., CO, USA). For continual monitoring of body temperature, rectal probe for small animals was used (AD Instruments Ltd., CO, USA). ECG was recorded before an ovariectomy (T0), before each *i.v.* administration of DOX (or vehiculum; T1–T5), and before the left ventricular catheterisation (T6).

ECG was analysed using LabChart 8 Pro ECG module (AD Instruments Ltd., CO, USA). Sixty seconds interval without significant artefacts was selected for the analysis. R peaks were automatically detected and heart rate (HR) was analysed.

### Left Heart Catheterisation

A week after the last dose of the *i.v.* treatment, heart catheterisation was performed using a Millar catheter (model SPR-738) connected to MPVS Ultra Pressure-Volume Unit (Millar Inc., TX, USA) and PowerLab Data Acquisition System (AD Instruments Ltd., CO, USA). Under isoflurane anaesthesia (5% induction, 2.5% maintenance), a catheter was inserted via the left carotid artery into the aortic root and subsequently into the left ventricle. Once positioned, pressure and volume characteristics were continuously recorded for 1 min. The mean arterial pressure (MAP) in the aorta and left ventricular indices dP/dt_max_ and dP/dt_min_ were evaluated using LabChart 8 Pro software (AD Instruments Ltd., CO, USA).

### Experiment Termination, Samples Collection

After completing heart catheterisation, the sample of anticoagulated blood was collected by intracardiac puncture. Blood sample was immediately centrifuged and obtained blood plasma was aliquoted and frozen at -80 °C until analysis. Subsequently, the rat was sacrificed by anaesthetic overdose and the heart was rapidly excised. The heart was washed from remaining blood by cold (4 °C) phosphate-buffered saline, weighed, and fixed in formalin for 24 h for histopathological analysis. Immediately after heart excision, thoracic aorta was isolated and placed into warm (37 °C) K-H sol. bubbled with 95% O_2_ + 5% CO_2_.

### Blood Plasma Analysis

The concentration of 4-HNE in plasma was determined using high-performance liquid chromatography (HPLC) with fluorescence detection. Prior to analysis, the samples underwent derivatisation and preliminary separation. The derivatisation procedure was based on a previously described method [47] with minor modification. Briefly, rat plasma was incubated with derivatisation reagent 1,3-cyclohexanedione at 60 °C for 1 h. Subsequently, the derivatised samples were cooled to room temperature, followed by addition of methanol for protein precipitation. The samples were then centrifuged. The resulting fluorescent derivatives were further purified by solid-phase extraction (Strata C18-U) and quantified by HPLC (Luna 3 μm C18, 100 × 3 mm) with fluorescence detection (excitation 380 nm; emission 445 nm).

### Histopathological Analysis of Myocardium

After formalin fixation, the heart was sectioned perpendicularly to the long axis and routinely processed into formalin-fixed, paraffin-embedded tissue blocks. Histopathological examination was performed using haematoxylin-eosin staining. Standard histopathological evaluation was conducted in accordance with the recommendations of the European Society of Cardiology [48] by an experienced pathologist, with particular attention to features of myocardial remodelling.

### Isolated Aortal Ring

**Fig. 2:**
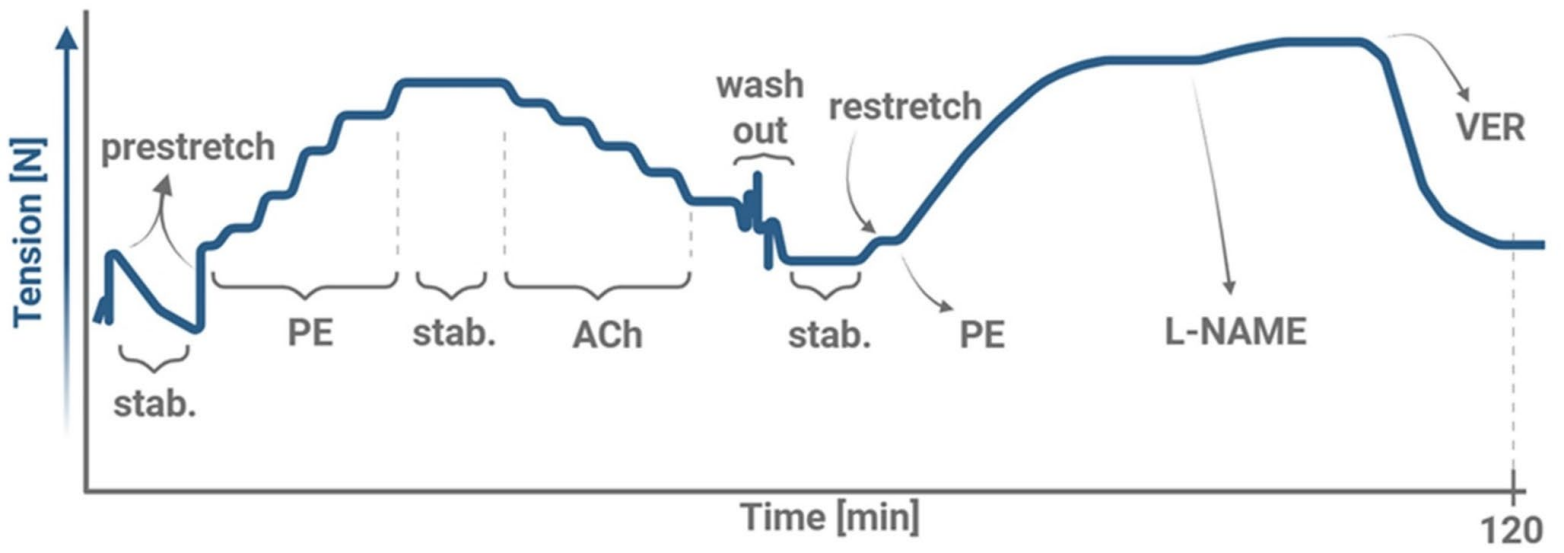
Experimental Protocol of Isolated Aortal Ring. The graph illustrates the time course of tension changes in isolated aortal ring during the experiment. Phases of experiment: stab. – stabilisation, administration of phenylephrine (PE), acetylcholine (ACh), L-N-Nitro arginine methyl ester hydrochloride (L-NAME), verapamil (VER)

Immediately after the isolation, the thoracic aorta was cleansed from blood and surrounding tissues. The thin (2 mm) aortal ring was cut and fixed on the single-chamber myograph (DMT751-Mini-Tobs, Denmark) using stainless steel hooks. The ring was immersed in fresh K-H sol. (37 °C, bubbled with 95% O_2_ + 5% CO_2_) and tension was continuously recorded throughout the whole experiment (Fig. 2). After setting the initial tension (10 mN; prestretch), the aortal ring was stabilised (15 min) and prestretched again to 10 mN. The physiological functions of the ring were verified by cumulative dose–response of PE and then ACh, both in gradually increasing doses (0.1 µM, 0.3 µM, 1 µM, 3 µM, 10 µM; administered in 3 min interval each). A stabilisation phase (15 min) was inserted between the administration of PE and ACh. Then, the aortal ring was washed out four times by the K-H sol. (37 °C, 95% O_2_ + 5% CO_2_). If necessary, the passive tension of the ring was set again (10 mN, restretch). Subsequently, a single dose of PE (2 µM) was administered. After 10 min, L-NAME (300 µM) was added (20 min). Finally, VER (11 µM) was added (10 min). The effect of L-NAME on PE-induced tension was expressed as a Phenylephrine Contraction Ratio (PECR), e.g., the ratio between the tension after L-NAME administration and the tension induced by PE alone. The PECR serves as a measure of alteration in nitric oxide synthase (NOS) function [49].

### Statistical Analyses

Statistical analyses were performed using GraphPad Prism^®^ version 10 (GraphPad Software, Inc., CA, USA). Descriptive statistics (mean, median, standard deviation) and the Shapiro–Wilk normality test were calculated for each data set. For the comparison of selected physiological or biochemical parameters among experimental groups, ordinary one-way analysis of variance (ANOVA) was used. When significant differences were detected, post hoc multiple comparisons were performed using Tukey’s test. For repeated measures data, such as electrocardiographic and echocardiographic parameters assessed at multiple time points, a mixed-effects model with restricted maximum likelihood estimation (REML) was applied. This approach accounted for missing values and individual variability over time. For pharmacological experiments involving isolated aortic rings, dose–response curves were constructed and analysed using nonlinear regression with a four–parameter logistic model (log[agonist] vs. response–variable slope). Comparisons between dose–response curves were performed by evaluating differences in curve parameters using extra sum–of–squares F tests. To assess the effects of treatment and substance on vascular tension, a two-way ANOVA was conducted, followed by Tukey’s multiple comparisons test. All data mentioned in the text are presented as mean ± standard deviation (SD), and p-values less than 0.05 were considered statistically significant.

## Results

### Fractional Shortening

**Fig. 3:**
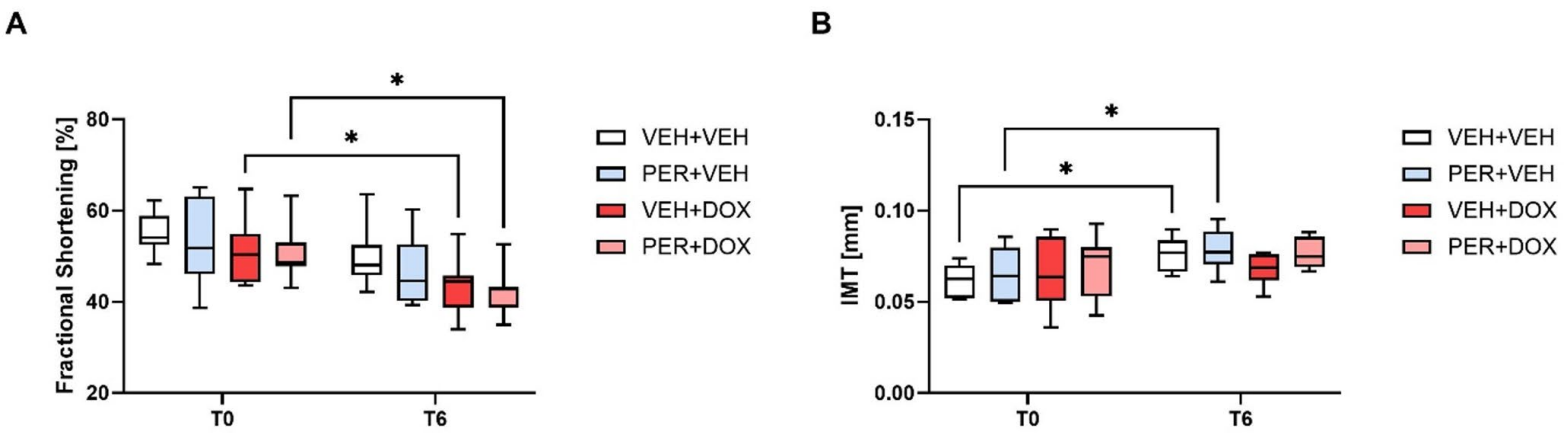
Fractional Shortening of the Left Ventricle (**A**) and Intima-Media Thickness (IMT; **B**). Data are presented as box-and-whiskers plots, showing the median (line within the box), interquartile range (box), and minimum and maximum values (whiskers). Experimental groups: VEH + VEH (negative controls; *n* = 8), PER + VEH (perindopril-treated rats; *n* = 8), VEH + DOX (doxorubicin-treated rats; *n* = 7), PER + DOX (simultaneously perindopril- and doxorubicin-treated rats; *n* = 8), * – *p* < 0.05

At T0, the mean fractional shortening of the left ventricle was higher than 50% in all groups (Fig. 3A). Significant decrease in fractional shortening was detected at T6 in the group VEH + DOX (43.53 ± 6.54%; *p* = 0.0499, compared with T0) and PER + DOX (42.27 ± 5.13%; *p* = 0.0241, compared with T0). There was no significant change in the fractional shortening in the VEH + VEH and PER + VEH groups at T6. The differences among the groups at T6 were not statistically significant. No significant differences were detected in the left ventricular wall thickness and intraventricular septal thickness among the groups. For more details, see Supplementary Information (Fig. S1).

### Intima-Media Thickness

At baseline (T0), no significant differences in IMT were observed among the experimental groups (Fig. 3B). By the end of the study (T6), a significant increase in IMT was detected in both the VEH + VEH and PER + VEH groups compared to their respective T0 values (for VEH + VEH: 0.062 ± 0.009 mm at T0 vs. 0.076 ± 0.009 mm at T6, *p* = 0.0354; for PER + VEH: 0.065 ± 0.015 mm at T0 vs. 0.079 ± 0.012 mm at T6, *p* = 0.0401). No significant changes were observed in the VEH + DOX and PER + DOX groups over time. Although the VEH + DOX group exhibited a trend toward lower IMT at T6 compared with the other groups (0.068 ± 0.009 mm in VEH + DOX; 0.076 ± 0.009 mm in VEH + VEH; 0.079 ± 0.012 mm in PER + VEH; 0.077 ± 0.008 mm in PER + DOX), this difference did not reach statistical significance.

### Heart Rate

At T0, no significant difference in the HR was detected among the groups (396.8 ± 32.2 bpm vs. 417.2 ± 34.9 bpm vs. 432.5 ± 32.0 bpm vs. 422.0 ± 32.2 bpm, for VEH + VEH vs. PER + VEH vs. VEH + DOX vs. PER + DOX, respectively). One week of PER administration (T1) led to a significant increase in the HR in the PER + VEH group compared with the VEH + VEH (control) group (442.5 ± 21.8 bpm vs. 408.3 ± 24.1 bpm; *p* = 0.0447). The HR of the VEH + DOX group decreased significantly at T6 (373.6 ± 26.7 bpm) compared with T0 (432.5 ± 32.0 bpm; *p* = 0.0078). The same downtrend was observed in the PER + DOX group (422.0 ± 32.2 bpm vs. 354.4 ± 22.7 bpm, for T0 vs. T6; *p* = 0.0038). At the end of follow-up (T6), there was a significant difference in the HR between PER + VEH and PER + DOX (406.2 ± 35.8 bpm vs. 354.4 ± 22.7 bpm; *p* = 0.0218). Nevertheless, no significant difference was observed between VEH + DOX and PER + DOX at T6 (Fig. S2).

### Mean Arterial Pressure, dP/dtₘₐₓ, dP/dt_min_

MAP changes were insignificant among the groups. The control group (VEH + VEH) showed the highest MAP values of 75.4 ± 22.5 mmHg. Administration of PER in the PER + VEH group resulted in a slight decrease in MAP (66.7 ± 23.4 mmHg). The VEH + DOX group showed a slightly lower MAP than VEH + VEH (70.0 ± 14.4 mmHg). The lowest values were recorded in PER + DOX, where MAP reached 62.2 ± 8.1 mmHg (Fig. S3).

The maximal rate of pressure increase in the left ventricle (dP/dtₘₐₓ) was insignificantly higher in VEH + VEH and PER + VEH groups (6271 ± 1977 mmHg/s and 5958 ± 2908 mmHg/s, respectively) than in VEH + DOX and PER + DOX groups (5539 ± 1124 mmHg/s and 4897 ± 964 mmHg/s, respectively). For more details, see Supplementary Information (Fig. S4).

Analysis of maximal rate of left ventricular pressure decline (dP/dt_min_) reveals insignificant differences among the groups. Mean values were in the range from  -6158 to -5233 mmHg/s (Fig. S5).

### 4-hydroxy-2-nonenal

**Fig. 4:**
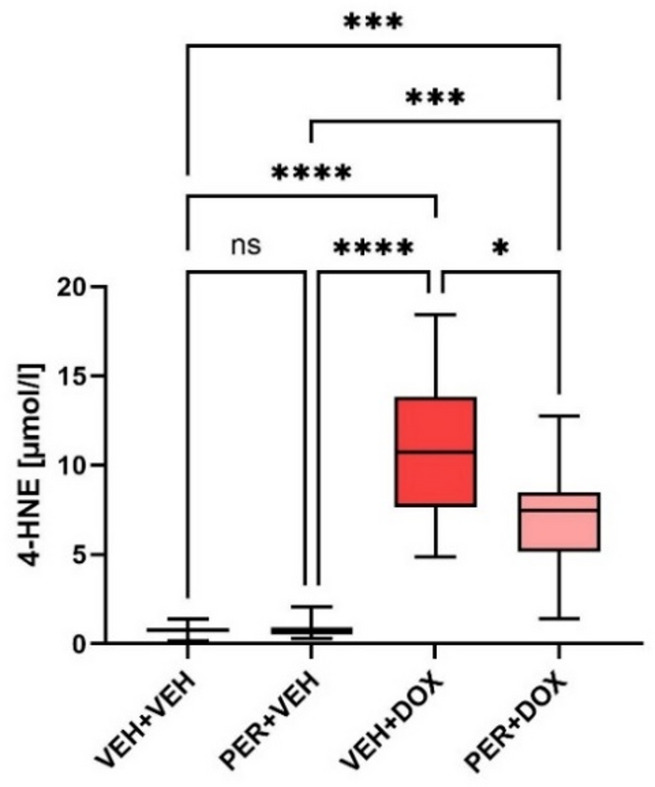
The Plasmatic Levels of 4-Hydroxy-2-Nonenal (4-HNE). Data are presented as box-and-whiskers plots, showing the median (line within the box), interquartile range (box), and minimum and maximum values (whiskers). Experimental groups: VEH + VEH (negative controls; *n* = 8), PER + VEH (perindopril-treated rats; *n* = 8), VEH + DOX (doxorubicin-treated rats; *n* = 7), PER + DOX (simultaneously perindopril- and doxorubicin-treated rats; *n* = 8), ns – not significant, * – *p* < 0.05, *** – *p* < 0.001, **** – *p* < 0.0001

In the VEH + VEH and PER + VEH groups, the levels of 4-HNE were very low (0.765 ± 0.334 µmol/l and 0.818 ± 0.538 µmol/l, respectively; Fig. 4). In the VEH + DOX group, a significant increase was observed compared with the VEH + VEH group (11.020 ± 4.388 µmol/l; *p* < 0.0001). In the PER + DOX group, a significant decrease was found compared with the VEH + DOX group (7.199 ± 3.271 µmol/l; *p* = 0.0473).

### Histopathological Analysis of Myocardium

Histopathological analysis of the heart muscle revealed no significant structural changes in the VEH + VEH group. In the PER + VEH group, focal interstitial remodelling was detected in one animal (out of 8). In 4 animals from the VEH + DOX group (out of 7), focal interstitial remodelling was detected. No significant structural changes in the PER + DOX group were detected. Representative photomicrographs of the myocardial samples are presented in Supplementary Information (Fig. S6).

### Isolated Aortal Ring Tension

PE and ACh induced the expected changes in aortal ring tension, e.g., an increase with PE and a decrease with ACh. No significant differences in dose–response curves for either PE or ACh were observed among the groups.

**Fig. 5:**
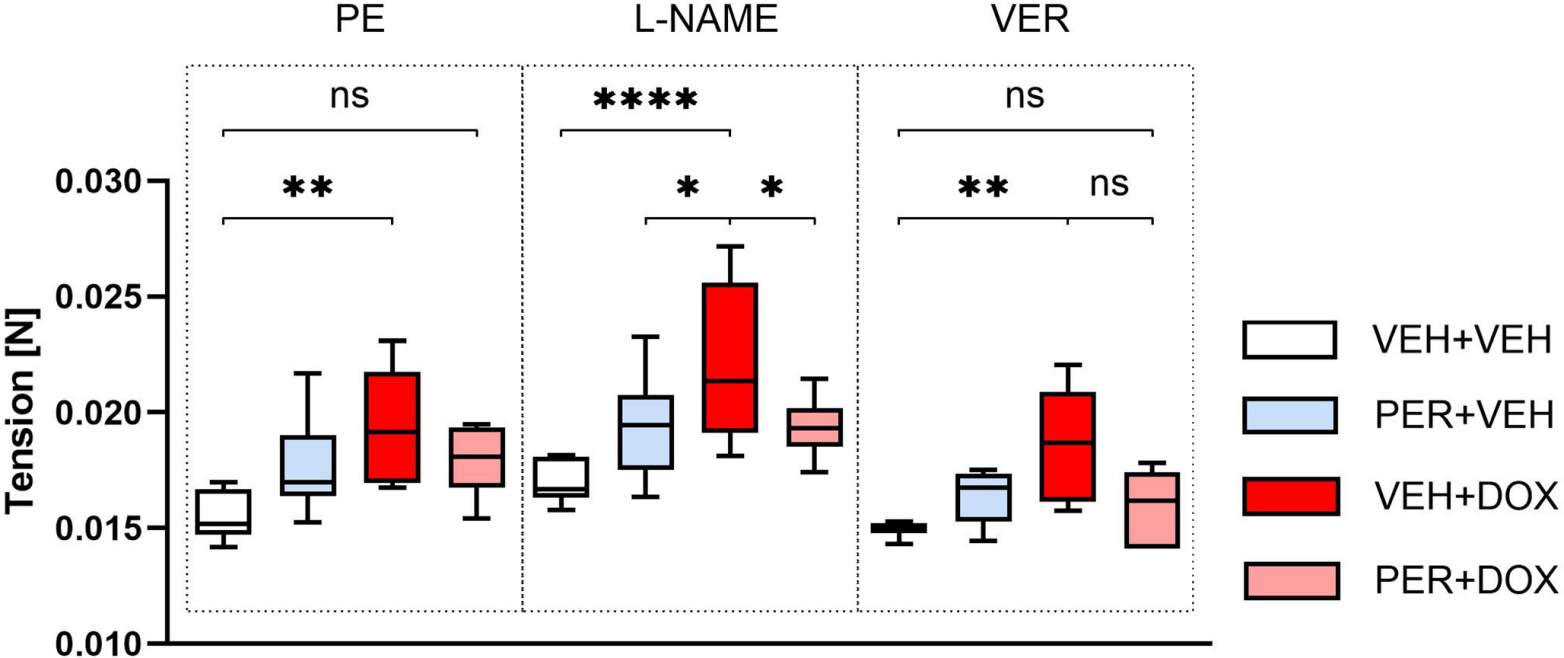
Changes in Tension of the Aortal Ring after Administration of a Single Dose of Phenylephrine (PE), L-N-Nitro Arginine Methyl Ester Hydrochloride (L-NAME) and Verapamil (VER). Data are presented as box-and-whiskers plots, showing the median (line within the box), interquartile range (box), and minimum and maximum values (whiskers). Experimental groups: VEH + VEH (negative controls; *n* = 8), PER + VEH (perindopril-treated rats; *n* = 8), VEH + DOX (doxorubicin-treated rats; *n* = 7), PER + DOX (simultaneously perindopril- and doxorubicin-treated rats; *n* = 8), ns – not significant, * – *p* < 0.05, ** – *p* < 0.01, **** – p < 0.0001

A single dose of PE (2 µM) induced slightly higher tension in the PER + VEH group (17.65 ± 2.03 mN) compared with the VEH + VEH group (15.61 ± 1.08 mN, *p* = 0.1530; Fig. 5). Similarly, PER + DOX showed slightly increased tension (17.93 ± 1.44 mN, *p* = 0.0814 vs. VEH + VEH). VEH + DOX exhibited significant increase compared with VEH + VEH (19.46 ± 2.38 mN, *p* = 0.0012).

A single dose of L-NAME (300 µM) induced an increase in tension across all groups (Fig. 5; Table 1). Significant differences were found between VEH + DOX and VEH + VEH (22.27 ± 3.35 mN vs. 17.01 ± 0.89 mN, *p* < 0.0001), between VEH + DOX and PER + VEH (22.27 ± 3.35 mN vs. 19.38 ± 2.18 mN, *p* = 0.0177), and between PER + DOX and VEH + DOX (19.46 ± 1.30 mN vs. 22.27 ± 3.35 mN, *p* = 0.0287).

A single dose of VER (11 µM) induced a significant decrease in tension across all groups (Fig. 5; Table 1). The VEH + DOX group exhibited significantly higher tension compared with the VEH + VEH group (18.46 ± 2.43 mN vs. 14.95 ± 0.33 mN, *p* = 0.0037). However, no significant difference was found between the PER + DOX group (15.90 ± 1.59 mN) and the VEH + VEH group following VER administration.

**Table 1:** Pairwise comparisons of aortal ring tension following sequential administration of phenylephrine (PE), L-N-Nitro arginine methyl ester hydrochloride (L-NAME) and verapamil (VER)

Group	PE vs. L-NAME	PE vs. VER	L-NAME vs. VER
VEH + VEH	0.3363 (ns)	0.7863 (ns)	0.1006 (ns)
PER + VEH	0.1526 (ns)	0.3515 (ns)	0.0046 (**)
VEH + DOX	0.0154 (*)	0.5720 (ns)	0.0007 (***)
PER + DOX	0.2502 (ns)	0.0923 (ns)	0.0016 (**)

Values represent adjusted p-values from Tukey’s multiple comparisons test, performed following two-way ANOVA

Experimental groups: VEH + VEH (negative controls; *n* = 8), PER + VEH (perindopril-treated rats; *n* = 8), VEH + DOX (doxorubicin-treated rats; *n* = 7), PER + DOX (simultaneously perindopril- and doxorubicin-treated rats; *n* = 8)

A p-value < 0.05 was considered statistically significant. Significance levels are indicated in parentheses (ns – not significant; * – *p* < 0.05; ** – *p* < 0.01; *** – *p* < 0.001)

**Fig. 6:**
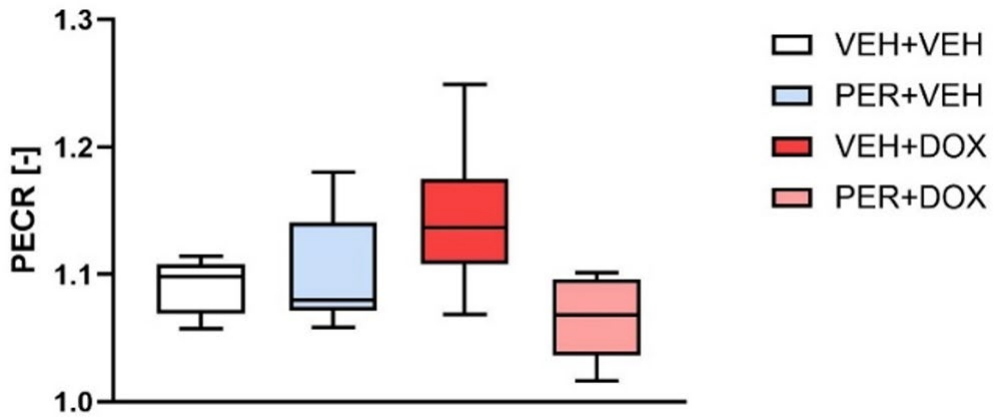
Phenylephrine Contraction Ratio (PECR). PECR was calculated as the ratio of vascular tension following administration of L-N-Nitro arginine methyl ester hydrochloride (L-NAME) to the tension induced by phenylephrine (PE) alone. This ratio serves as an indicator of alteration in nitric oxide synthase (NOS) function [49]. Data are presented as box-and-whiskers plots, showing the median (line within the box), interquartile range (box), and minimum and maximum values (whiskers). Experimental groups: VEH + VEH (negative controls; *n* = 8), PER + VEH (perindopril-treated rats; *n* = 8), VEH + DOX (doxorubicin-treated rats; *n* = 7), PER + DOX (simultaneously perindopril- and doxorubicin-treated rats; *n* = 8)

In the VEH + VEH group, the PECR was 1.091 ± 0.021. In the PER + VEH group, PECR values were comparable to VEH + VEH (1.099 ± 0.044). The VEH + DOX group exhibited a non-significant increase in PECR (1.142 ± 0.057), whereas the PER + DOX group showed a slight, non-significant decrease (1.065 ± 0.031). Although these data suggest observable trends, statistical analysis revealed no significant differences in PECR among the groups (Fig. 6).

## Discussion

Anthracyclines, particularly DOX, remain a cornerstone of chemotherapy for a wide range of malignancies. Despite their proven efficacy, these agents are associated with significant cardiovascular toxicity, which poses a major challenge in long-term cancer care [7]. DOX-induced cardiotoxicity is well documented, but its vascular effects have received comparatively less attention. As cancer survival rates improve, understanding and mitigating the cardiovascular consequences of chemotherapy has become increasingly important.

The risk of DOX-induced cardiovascular damage is influenced by cumulative dose, patient age, and pre-existing conditions such as metabolic syndrome and cardiovascular disease [34]. While clinical protocols attempt to limit cardiotoxicity through dose restrictions and symptomatic management [7, 50, 51], there are currently no evidence-based strategies specifically aimed at preventing vascular injury. Pharmacological interventions such as RAAS modulators (e.g., ACEIs), β-blockers, calcium channel blockers, and diuretics are commonly used to manage cardiovascular symptoms, but their protective role during chemotherapy remains under investigation. Although several agents – such as dexrazoxane [52, 53], pigment epithelium-derived factor [54], thrombopoietin [55], and various phytochemicals [56, 57] – have shown potential in preclinical studies, their translation into clinical practice has been limited due to inconsistent efficacy and safety concerns [18, 45, 46, 58–61].

Although TNBC is one of the clinical contexts where DOX is frequently used, and its aggressive nature often necessitates high-dose anthracycline therapy, the vascular risks associated with DOX extend beyond TNBC and affect a broad spectrum of cancer patients. In our study, we aimed to model a clinically relevant scenario [45] by using ovariectomized female rats, reflecting the increased cardiovascular vulnerability caused by decreased gonadal hormones levels in peri- and postmenopausal women undergoing chemotherapy [34, 62, 63].

In our study, we employed a clinically relevant DOX treatment protocol, consisting of bolus *i.v.* dose administered once a week for five consecutive weeks [25]. Although DOX is typically administered every three weeks in human patients, the shorter lifespan and accelerated metabolism of rats justify a condensed dosing schedule. The *i.v.* route was selected to closely mimic clinical practice, in contrast to the more commonly used intraperitoneal administration in animal studies. While intraperitoneal injection is technically simpler, it introduces variability in pharmacokinetics and carries risks such as peritoneal inflammation and pain, which may contribute to inconsistencies observed in previous studies [64].

Our retrospective clinical study indicated that peri- and postmenopausal women face a higher incidence of cardiovascular diseases, with hypertension being the most prevalent [45]. Among the most frequently prescribed medications in this population are ACEIs [45], which target the RAAS. PER, a well-tolerated ACEI, is commonly used either as monotherapy or in combination with other antihypertensives, such as calcium channel blockers [45, 65]. In clinical settings, PER is typically administered once a day. Accordingly, in our experimental design, PER was given *p.o.* once a day, with the dose adjusted to the animals’ body weight (1 mg/kg/day) to reflect clinical dosing practices.

In standard anthracycline-based chemotherapy protocols, cardiovascular monitoring typically includes ECG and ECHO before and after treatment, with blood pressure measured prior to each DOX dose. In our experimental setup, ECG and ECHO were recorded at baseline (T0) and at the end of the study (T6), with the final ECHO performed one day after the last DOX dose. Additionally, vascular ultrasound was conducted, which is not routinely performed in clinical oncology but critical for evaluating vascular toxicity in this context. ECG recordings were also obtained prior to each DOX administration. Blood pressure, however, could not be measured repeatedly due to technical limitations. Non-invasive tail-cuff methods, while standard in rodents, would have interfered with repeated *i.v.* access. Therefore, to obtain hemodynamic data, catheterization of the aorta and left ventricle was performed at the end of the experiment.

The observed decline in fractional shortening in both DOX-treated groups (VEH + DOX and PER + DOX) at T6 confirms the cardiotoxic potential of DOX, consistent with previous reports of impaired systolic function following anthracycline exposure. PER co-treatment did not prevent this reduction, suggesting that its protective effect may not extend to preserving contractile function under the conditions tested. It is important to emphasise that ECHO at T6 was performed one day after the final DOX dose; therefore, the measured fractional shortening likely reflects the acute effects of DOX rather than the subchronic toxicity [66].

Despite the lack of significant intergroup differences in HR at baseline (T0), PER administration alone (PER + VEH) transiently increased HR at T1, possibly reflecting compensatory autonomic modulation. In contrast, both DOX-treated groups exhibited a significant HR reduction at T6, which may indicate a progressive cardiac dysfunction. The lower HR in the PER + DOX group compared with PER + VEH at T6 further supports the idea that DOX-induced cardiotoxicity is only partially mitigated by PER.

MAP and ventricular pressure dynamics (dP/dtₘₐₓ and dP/dt_min_) did not differ significantly among groups, although a trend toward reduced MAP and contractility parameters in the VEH + DOX and PER + DOX groups may reflect cumulative cardiovascular compromise.

Myocardial tissue samples were collected to evaluate the potential histopathological changes associated with DOX administration. The absence of significant histopathological signs of myocardial injury is consistent with ECHO findings, where no changes in left ventricular wall thickness were detected. The lack of structural signs of myocardial injury and remodelling suggests that the experimental model was appropriately designed, particularly with respect to the DOX dosage, administration frequency, and treatment duration.

To evaluate DOX-induced vascular effects, thoracic aortal rings were explanted, and vascular reactivity was studied. Isolated aortal ring is an established model for testing vessel reactivity, bridging the gap between in vitro testing and in vivo models. It offers greater clinical relevance than in vitro assays, while being more time- and cost-efficient than in vivo studies, making it an ideal choice for preclinical drug testing. Moreover, it allows to evaluate endothelial or VSM functions by discretely affecting their specific signalling pathways [67]. Various methodological approaches differ in the way of aortal ring preparation, solution composition, temperature, initial passive tension, overall protocol, and its duration [68]. For instance, while several studies demonstrated that in cold solution and with prolonged storage the aortal ring keeps its qualities [69–71], Langen et al. emphasise the importance of time schedule, since NO bioavailability decreases significantly over time in this model [72]. As a result, the reported characteristics of the isolated aorta vary significantly in various studies.

In designing our experimental protocol, we were inspired by several previously published protocols [37, 42, 49, 68, 71, 73–76]. The experimental setup was standardized to ensure reproducibility, including consistent timing [72], use of the same aortal segment and initial passive tension of 10 mN [77–80], and strictly controlled solution conditions. PE, ACh, L-NAME, and VER were used to assess vascular reactivity.

After stabilization and passive tension setting, a selective α_1_ adrenoreceptor agonist PE was applied in gradually increasing doses in order to evaluate the vasoconstrictive capacity of the aortal ring with intact endothelium. Subsequently, a muscarinic receptor agonist ACh was added in order to assess the endothelial functions, since it induces endothelium-dependent vasodilation primarily by NO release. Isolated aortal rings from all experimental groups responded appropriately to both vasoactive agents, confirming tissue viability.

After washout, stabilisation and passive tension setting, a single dose of PE (2 µM) was used in order to elicit maximal vasoconstriction [76]. The significant tension differences between the VEH + VEH and VEH + DOX groups indicated DOX-induced vascular impairment. In contrast, the absence of significant differences between VEH + VEH and PER + DOX groups suggests a protective effect(s) of PER against DOX-induced vascular toxicity.

To further explore endothelial involvement in regulating VSM contraction and tone, NOS inhibitor L-NAME was administered. In other words, inhibition of NOS by L-NAME excluded the impact of NO in modulating vascular tone. The toxic effect(s) of DOX were again reflected in significant tension differences between the VEH + VEH and VEH + DOX. The protective effect of PER against DOX-induced vascular toxicity is directly demonstrated by the significantly reduced tension observed in the PER + DOX compared with VEH + DOX.

In order to observe real time impact of NOS inhibition on PE-induced tension development, PECR was calculated. This simple and species-independent index reflects NOS function directly, revealing NOS-dependent kinetic changes in endothelial and VSM function. Although PECR values did not reach statistical significance across groups, the observed trends were consistent with other findings. The lack of significance may be attributed to differences in PE and L-NAME concentrations compared to the original protocol by Jin et al. [49].

Finally, L-type Ca^2+^ channel antagonist VER was added to induce vasodilation by reducing intracellular free Ca^2+^ concentration. Its administration following L-NAME exposure helped to isolate the contribution of Ca^2+^ channels to VSM contraction [37, 68, 81, 82]. The results mirrored previous observations: DOX treatment led to increased vascular tension (significant tension differences between VEH + VEH and VEH + DOX), while PER co-treatment mitigated this effect, further supporting its protective role (insignificant tension differences between VEH + VEH and PER + DOX).

Our results suggest that the protective effect of PER against DOX-induced vascular toxicity is dominantly mediated by endothelium. Although this idea has been declared by several authors (reviewed by [40]), the full description of the underlying processes remains unclarified. Possible link among processes behind DOX-induced vascular toxicity represents oxidative stress. It is defined as an imbalance between the production of free radicals and the cell´s antioxidant defences, resulting in the excessive ROS generation, which in turn induces non-enzymatic lipid peroxidation. This process leads to the formation of a broad range of small reactive aldehydes [83, 84]. Among these, 4-HNE is a major aldehyde produced during peroxidation of membrane n-6 polyunsaturated fatty acids, mainly from arachidonic and linoleic acids [85–88]. Therefore, elevated 4-HNE levels generally reflect enhanced lipid peroxidation [85, 87], and as such, 4-HNE serves as suitable and broadly accepted marker of oxidative stress. Moreover, it is known to contribute to the development and progression of various pathologies, including metabolic diseases, neurodegenerative diseases, cardiovascular diseases, and cancer [87, 89]. Among others, ROS are known to trigger endothelial remodelling [40, 84, 90, 91], e.g., by activation of NF-κB signalling pathway [92]. This signalling pathway in endothelial cells brings about a thought-provoking idea [93]: angiotensin-converting enzyme (ACE) inhibition could be a key player in mitigating DOX-induced cardiovascular toxicity, because ACE: (1) increases the production of ROS (e.g., activates NADPH oxidase); (2) accelerates NO inactivation and inhibits NO production via bradykinin degradation; (3) inhibits endothelial NOS and prostacyclin synthase. Thus, inhibition of ACE significantly lowers synthesis of ROS, increases level of NO, and activates NOS in endothelial cell [93–95]. Moreover, ACE promotes vascular inflammation via binding AT_1_ receptors and subsequent NF-κB-mediated pro-inflammatory responses in endothelial cells [96].

DOX has been reported to induce dose-dependent endothelial-to-mesenchymal transition, a process closely linked to oxidative stress and vascular remodelling [90, 97, 98]. In order to evaluate the oxidative stress burden in our experimental model, blood plasma samples were collected at T6 and analysed for 4-HNE, a well-established marker of lipid peroxidation and oxidative damage.

Quantitative comparison among the experimental groups revealed significant differences in 4-HNE levels. In the VEH + VEH and PER + VEH groups, 4-HNE concentrations remained low, indicating minimal oxidative stress. In contrast, DOX administration led to a marked increase in 4-HNE levels in the VEH + DOX group, confirming the oxidative effect of DOX. Importantly, co-treatment with PER significantly attenuated this increase, suggesting that PER may mitigate DOX-induced oxidative stress, likely through its antioxidant and endothelial-protective mechanisms. These findings support the hypothesis that oxidative stress plays a central role in DOX-induced vascular toxicity and that ACEI may offer a viable strategy to counteract this effect.

To assess structural vascular remodelling, we measured carotid artery IMT at baseline (T0) and at the end of the study (T6). No significant differences were observed among the groups at T0, confirming comparable baseline vascular status. By T6, a significant increase in IMT was detected in both the VEH + VEH and PER + VEH groups, likely reflecting age-related or hormonal remodelling over the course of the experiment. In contrast, IMT values in the DOX-treated groups (VEH + DOX and PER + DOX) remained stable and appeared slightly lower than those in the non-DOX groups, although the differences did not reach statistical significance.

This pattern is particularly interesting in the context of the experimental design. All animals were ovariectomized shortly after baseline measurements, inducing oestrogen deficiency, a known driver of vascular remodelling and IMT progression [99]. The observed IMT increase in the non-DOX groups may therefore reflect early post-ovariectomy vascular changes. Conversely, the absence of IMT progression in the DOX-treated groups could be attributed to DOX-induced endothelial dysfunction, which may impair the normal adaptive thickening response of the vascular wall. This interpretation is further supported by the relatively short follow-up period (five weeks) and the fact that T6 IMT measurements were taken just one day after the final DOX dose, capturing predominantly acute effects.

## Limitations and Future Perspectives

Although many efforts are being made to establish an ideal animal model to imitate the clinical practice, there are still some limitations. The present study was designed with respect to the results from our previous clinical retrospective study of patients diagnosed with TNBC at Masaryk Memorial Cancer Institute. Thus, a crucial step in designing our present study was a complete list of their pharmacotherapy. However, it is known that in clinical practice misleading information and non-compliance from patients may subsequently affect the clinical trials [100]. Obtaining the information about the actual medication and other supportive substances (e.g., herbs [93]) used by the patient is important.

Despite all efforts to develop an anaesthetic that would not affect cardiovascular parameters, it has still not been invented. Isoflurane is one of the gentler/safer anaesthetics, but it still affects the cardiovascular system [101]. Use of anaesthesia may affect the measured ECG parameters. This drawback is minimized by the same approach in all experimental groups.

For the experiment, an aortal ring was used, which is a model of elastic artery. In order to get a more comprehensive view of the reactivity of a muscular vessel type, peripheral (e.g., femoral or renal [102–104]) artery can be employed.

Although our study included histopathological assessment of myocardial tissue, we did not perform a corresponding microscopic evaluation of the aorta or other large arteries, which limits our ability to directly corroborate functional changes in aortic ring tension with structural evidence such as inflammation, fibrosis, or endothelial integrity. Such analysis could have provided important insights into vascular remodelling and endothelial dysfunction, complementing the IMT measurements and ultrasound findings. Future studies should incorporate detailed histological examination of the arterial wall to strengthen mechanistic interpretation and validate functional outcomes.

This study was conducted using young, healthy female rats, which may not fully represent the clinical population typically affected by chemotherapy-related cardiovascular toxicity. Future research should consider including older and/or hypertensive animals to better reflect age-related and comorbid conditions. The inclusion of male rats is also essential, as the incidence of breast cancer in men – although still lower than in women – has nearly doubled over the past 30 years, rising from 0.7 per 100,000 in 1991–1995 to 1.33 per 100,000 in 2016–2020 [105]. Another reason for repeating the study on male animals is the fact that male and female gonadal hormones exhibit different effect on RAAS activity [63, 106]. Moreover, the specific aspects of breast cancer in transgender individuals should be considered [107]. Hypertension models may include both genetically predisposed strains and induced models, such as the DOCA/salt protocol [108]. Additionally, the use of immunodeficient (e.g., nude) rats with induced tumours could provide a more clinically relevant setting. Finally, future studies should explore the effects of co-administration of DOX with targeted therapies (e.g., pembrolizumab) and investigate sex-related differences in treatment response.

## Conclusion

Our findings suggest that while DOX clearly impairs vascular function and increases oxidative stress, its impact on structural remodelling may be more complex, potentially involving suppression of normal vascular adaptation. The apparent stabilising effect of PER on IMT in the PER + DOX group further supports its protective role in preserving vascular integrity. The studies focused on the mechanism(s) of DOX-induced vascular toxicity are scarce and the studies on ACEIs in this context are virtually absent, although ACEIs belong to the most widely used and economically accessible medications for the treatment of cardiovascular diseases. Our study provides evidence that PER, a well-established ACEI, offers protective effects against DOX-induced vascular toxicity. This was demonstrated through functional vascular assessments, reduced oxidative stress markers (namely 4-HNE), and preserved endothelial responses. Although structural changes in the vascular wall measured by IMT were less pronounced, the absence of IMT progression in DOX-treated animals may reflect impaired vascular remodelling due to endothelial dysfunction. This effect seems to be counteracted by PER administration.

Taken together, these findings support the consideration of PER as a prophylactic agent in patients undergoing anthracycline-based chemotherapy. Further research is warranted to validate these results in long-term studies and clinical settings, and to explore the broader potential of RAAS modulation in cardio-oncology.

## Supplementary Information

Below is the link to the electronic supplementary material.

Supplementary Material 1

## Data Availability

The data that support the findings of this study are not publicly available due to ongoing analyses and the intention to publish additional results in future publications. The data are stored in a controlled-access repository at Masaryk University and are available from the corresponding author upon reasonable request.
