# Supplementary material for: Role of Perindopril in Mitigating Doxorubicin’s Vascular Toxicity in a Rat Model
Source: Cardiovasc Toxicol. 2026 Jan 27;26(2):19. doi: 10.1007/s12012-026-10092-0 (PMC12847116; doi:10.1007/s12012-026-10092-0)
Supplement: Supplementary file 1 — Supplementary Material 1 [file 12012_2026_10092_MOESM1_ESM.pdf]

Role of Perindopril in Mitigating Doxorubicin's Vascular Toxicity in a Rat Model

Anna Marada[1], Tibor Stračina[1*], Filip Marhefka[1], Lucie Šůstková[1], Jaroslav Nádeníček[1], Jindra Smutná[2], Peter Scheer[3,4], Jana Hložková[3,4], Michal Hendrych[5], Christian Studenik[6], Hana Paulová[2], Marie Nováková[1#]

[1]Department of Physiology, Faculty of Medicine, Masaryk University, Brno, Czech Republic

[2]Department of Biochemistry, Faculty of Medicine, Masaryk University, Brno, Czech Republic

[3]Department of Pharmacology and Toxicology, Faculty of Pharmacy, Masaryk University, Brno, Czech Republic

[4]International Clinical Research Center, St. Anne's University Hospital Brno and Faculty of Medicine, Masaryk University, Brno, Czech Republic

[5]First Department of Pathology, Faculty of Medicine, Masaryk University and St. Anne's University Hospital Brno, Brno, Czech Republic

[6]Division of Pharmacology and Toxicology, Department of Pharmaceutical Sciences, University of Vienna, Vienna, Austria

Supplementary Information

This document provides supplementary material for the original article titled "*Role of Perindopril in Mitigating Doxorubicin's Vascular Toxicity in a Rat Model*" (DOI: 10.1007/s12012-026-10092-0). All included figures are directly related to the main text and are referenced in the corresponding sections of the article.

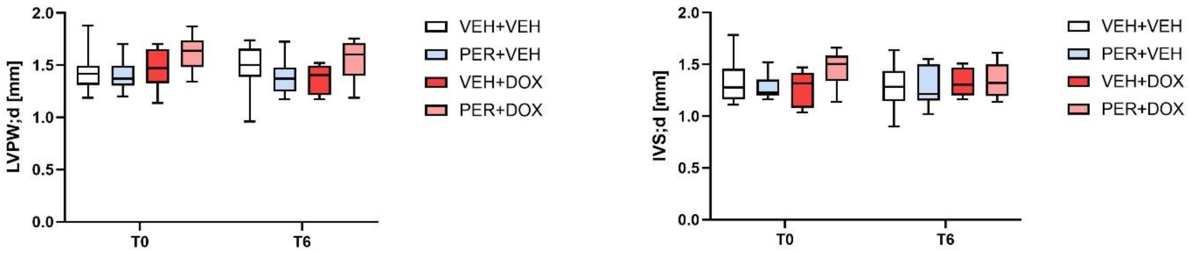

Figure S1: Thickness of left ventricular wall (LVW) and interventricular septum (IVS) in diastole. A high-frequency ultrasound imaging was performed using Vevo 2100 (FUJIFILM VisualSonics Inc., Toronto, Canada) at two time points: at the beginning of the experiment prior to ovariectomy (T0) and one day after the final dose of doxorubicin (T6). Animals were deeply anesthetized with isoflurane (2.5% for induction, 1–-1.5% for maintenance) and positioned on a heated platform. Diastolic left ventricular wall thickness and interventricular septum thickness were evaluated by transducer MS-201 in the parasternal long-axis view. Data are presented as box-and-whiskers plots, showing the median (line within the box), interquartile range (box), and minimum and maximum values (whiskers). Experimental groups: VEH+VEH (negative controls; n = 8), PER+VEH (perindopril-treated rats; n = 8), VEH+DOX (doxorubicin-treated rats; n = 7), PER+DOX (simultaneously perindopril- and doxorubicin-treated rats; n = 8). Statistical analysis was performed using a mixed-effects model with restricted maximum likelihood estimation (REML) in GraphPad Prism® version 10 (GraphPad Software, Inc., CA, USA). No statistically significant differences were detected among the groups.

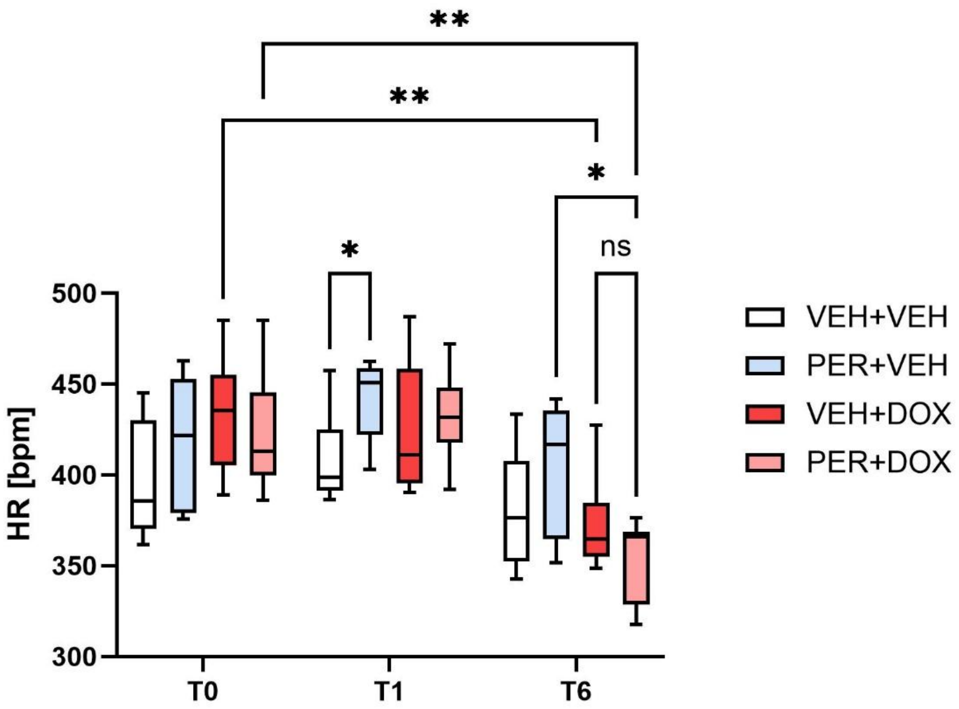

Figure S2: Heart rate of experimental animals. Heart rate was analysed using LabChart 8 Pro ECG module (AD Instruments Ltd., CO, USA) from a 60-second interval of one-lead ECG recorded using subcutaneous needle electrodes under isoflurane anaesthesia (5% induction, 2.5% maintenance). ECG recordings were obtained at three time points: before ovariectomy (T0), before the first intravenous administration of doxorubicin or vehiculum (T1), and before left ventricular catheterisation (T6). Data are presented as box-and-whiskers plots, showing the median (line within the box), interquartile range (box), and minimum and maximum values (whiskers). Experimental groups: VEH+VEH (negative controls; n = 8), PER+VEH (perindopril-treated rats; n = 8), VEH+DOX (doxorubicin-treated rats; n = 7), PER+DOX (simultaneously perindopril- and doxorubicin-treated rats; n = 8). Statistical analysis was performed using a mixed-effects model with restricted maximum likelihood estimation (REML) in GraphPad Prism® version 10 (GraphPad Software, Inc., CA, USA). Significance is indicated as: ns – not significant, * – $p < 0.05$, ** – $p < 0.01$.

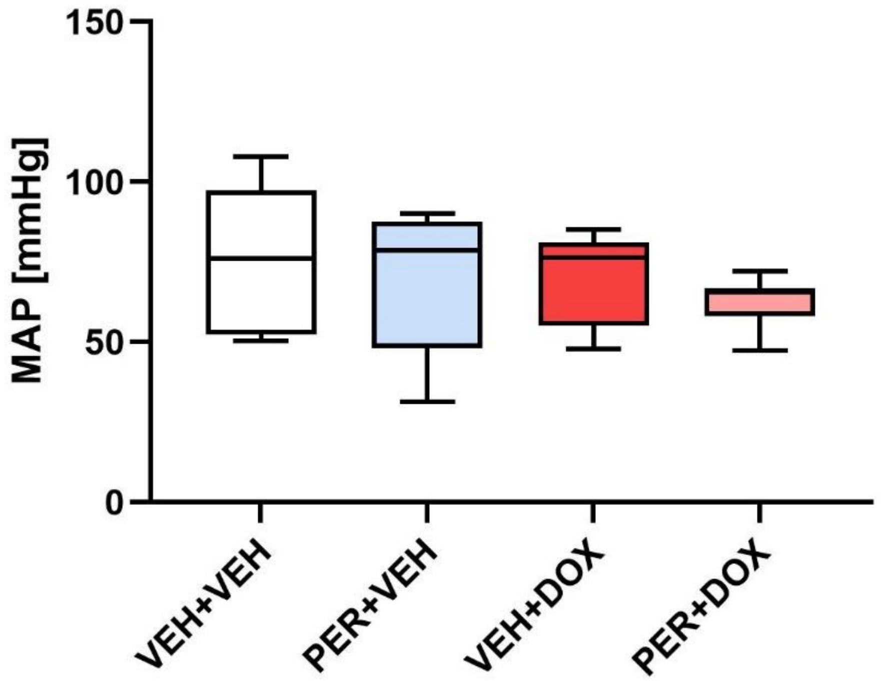

Figure S3: Mean arterial pressure (MAP). MAP was measured using a Millar catheter (model SPR-738) connected to MPVS Ultra Pressure-Volume Unit (Millar Inc., TX, USA) and a PowerLab Data Acquisition System (AD Instruments Ltd., CO, USA) under isoflurane anaesthesia (5% induction, 2.5% maintenance). MAP was evaluated in 60-second stable recording using LabChart 8 Pro software (AD Instruments Ltd., CO, USA). Experimental groups: VEH+VEH (negative controls; n = 8), PER+VEH (perindopril-treated rats; n = 8), VEH+DOX (doxorubicin-treated rats; n = 7), PER+DOX (simultaneously perindopril- and doxorubicin-treated rats; n = 8). Statistical analysis was performed using ordinary one-way ANOVA in GraphPad Prism® version 10 (GraphPad Software, Inc., CA, USA). No significant differences were detected among the groups.

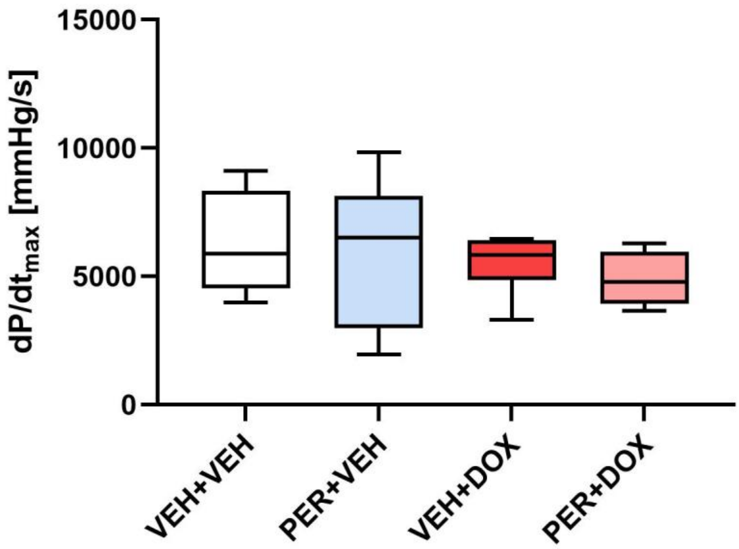

Figure S4: The maximal rate of pressure increase in the left ventricle (dP/dt$_{max}$). The dP/dt$_{max}$ index was calculated from stable recording of left ventricular pressure and volume using LabChart 8 Pro software (AD Instruments Ltd., CO, USA). Pressure-volume measurements were obtained with a Millar catheter (model SPR-738) connected to an MPVS Ultra Pressure-Volume Unit (Millar Inc., TX, USA) and a PowerLab Data Acquisition System (AD Instruments Ltd., CO, USA) under isoflurane anaesthesia (5% induction, 2.5% maintenance). Experimental groups: VEH+VEH (negative controls; n = 8), PER+VEH (perindopril-treated rats; n = 8), VEH+DOX (doxorubicin-treated rats; n = 7), PER+DOX (simultaneously perindopril- and doxorubicin-treated rats; n = 8). Statistical analysis was performed using ordinary one-way ANOVA in GraphPad Prism® version 10 (GraphPad Software, Inc., CA, USA). No significant differences were detected among the groups.

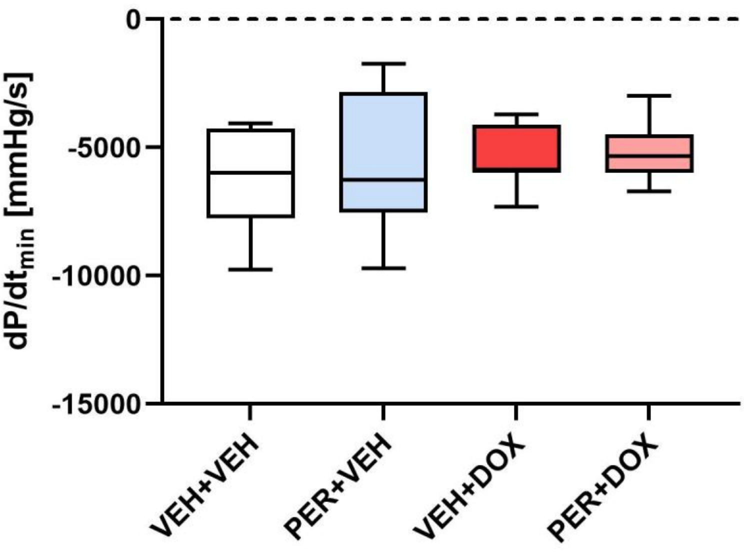

Figure S5: The maximal rate of pressure decline in the left ventricle (dP/dt$_{min}$). The dP/dt$_{min}$ index was calculated from stable recording of left ventricular pressure and volume using LabChart 8 Pro software (AD Instruments Ltd., CO, USA). Pressure-volume measurements were obtained with a Millar catheter (model SPR-738) connected to an MPVS Ultra Pressure-Volume Unit (Millar Inc., TX, USA) and a PowerLab Data Acquisition System (AD Instruments Ltd., CO, USA) under isoflurane anaesthesia (5% induction, 2.5% maintenance). Experimental groups: VEH+VEH (negative controls; n = 8), PER+VEH (perindopril-treated rats; n = 8), VEH+DOX (doxorubicin-treated rats; n = 7), PER+DOX (simultaneously perindopril- and doxorubicin-treated rats; n = 8). Statistical analysis was performed using ordinary one-way ANOVA in GraphPad Prism® version 10 (GraphPad Software, Inc., CA, USA). No significant differences were detected among the groups

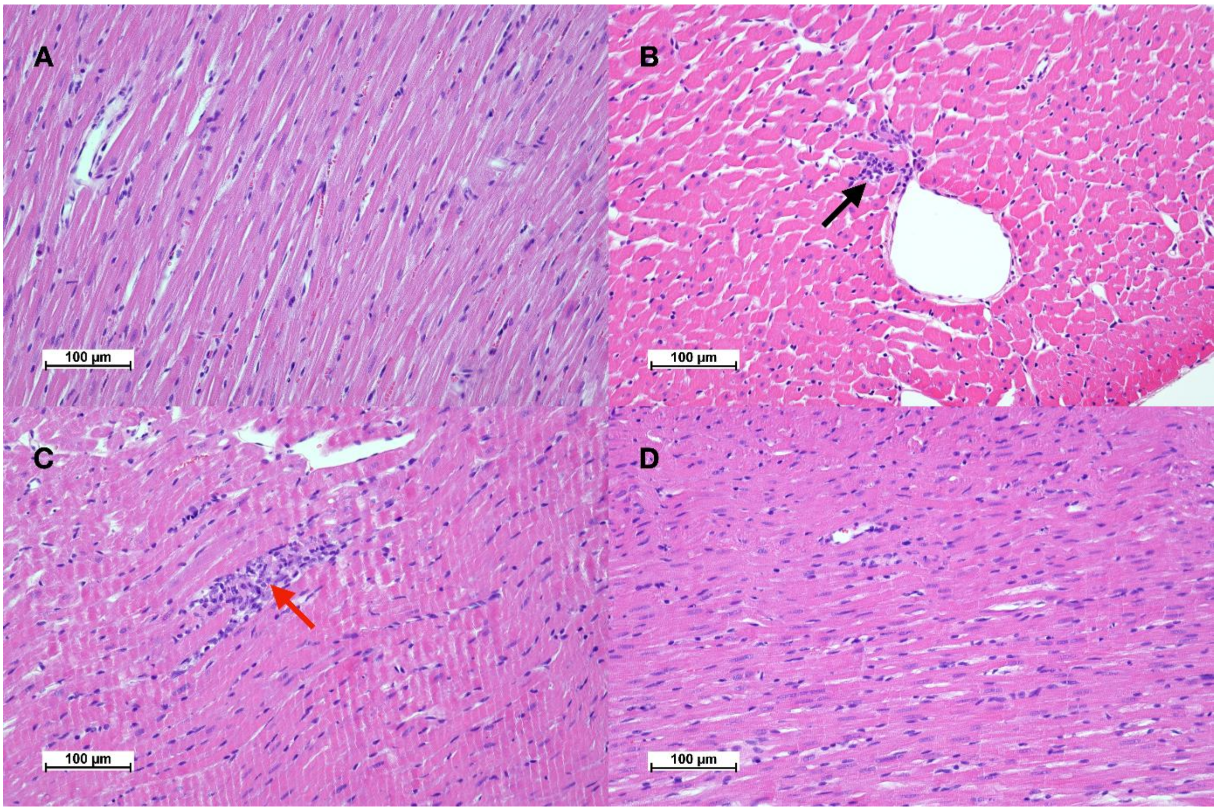

Figure S6: Representative photomicrographs of the myocardial samples. Myocardial tissue samples were routinely processed into formalin-fixed, paraffin-embedded tissue blocks. Histopathological assessment was performed using haematoxylin-eosin staining by an experienced pathologist, with particular attention to features of myocardial remodelling. No structural alterations were detected in VEH+VEH group (A). Focal cardiac remodelling was detected in one case of PER+VEH group in the vicinity of a vessel, as highlighted by the black arrow (B). In VEH+DOX group, interstitial remodelling is highlighted by the red arrow (C). While no structural changes were detected in PER+DOX group (D).
